# Amphetamine-induced Reverse Takotsubo Syndrome: Further Implications

**DOI:** 10.5811/cpcem.63004

**Published:** 2026-08-31

**Authors:** Kenan Yalta, Murat Gok

**Affiliations:** Trakya University, School of Medicine, Cardiology Department, Edirne, TÜRKİYE

Dear Editor:

In their recently published case report, Rabil et al^1^ describe a case of amphetamine-induced reverse takotsubo syndrome accompanied by arterial thrombosis at multiple sites in a young female patient.^1^ In this context, we would like to underscore further implications of amphetamine use, particularly in the context of takotsubo syndrome based on this interesting case.

First, atypical takotsubo syndrome variants have been attributed to high-risk clinical scenarios characterized by extreme levels of adrenergic discharge, potentially leading to malignant arrhythmogenesis, delayed takotsubo syndrome recovery, and coronary microvascular dysfunction.^2^ In this context, disproportionate sinus tachycardia on admission might also substantiate the presence of extreme adrenergic discharge in the patient.^1^ Beta blockers have been regarded as first-line agents for the management of sinus tachycardia, particularly in the setting of takotsubo syndrome episodes with supposedly extreme levels of adrenergic discharge (in the context of pheochromocytoma, etc).^3^ However, initiation of ivabradine might be deemed a safer alternative to beta blockers in takotsubo syndrome patients with compensatory sinus tachycardia and/or pulmonary edema.^3^ On the other hand, sinus tachycardia in the patient could be primarily attributed to metamphetamine impact,^1^ which might have been more effectively reversed with the use of sympatholytic agents including short-acting beta blockers. Intravenous ultra-short acting cardioselective beta blockers such as landiolol have been used for the management of various tachyarrhythmias even in the setting of left ventricular dysfunction (in reduced doses).^4^

Second, we hold the opinion that invasive coronary angiogram should have been performed in the patient on admission due to the overt ST-segment elevation pattern on electrocardiogram, existing high-risk clinical features, and low interTAK score, all of which might denote co-existing acute coronary syndrome.^3^ Mechanistically, excessive adrenergic discharge may elicit simultaneous evolution of takotsubo syndrome and spontaneous coronary artery dissection (SCAD), particularly in the female population.^5^ Moreover, SCAD may arise as a primary phenomenon that might subsequently lead to takotsubo syndrome evolution in the setting of extreme stressors.^5^ In this context, amphetamine use, besides its impact on the induction of arterial thrombus formation, was previously reported to induce SCAD as well as dissections in other arterial structures.^6^,^7^ Accordingly, we recommend the evaluation of coronary arteries and other arterial structures on further imaging modalities including computed tomography coronary angiogram in the post-discharge setting.

Finally, amphetamine use may also be associated with other forms of myocardial injury through various mechanisms including direct myocardial toxicity, coronary vasospasm, etc.^1^,^7^ Therefore, the patient may have incurred adverse myocardial remodeling and possible dilated cardiomyopathy in the long term due to the amphetamine-induced irreversible myocardial injury, potentially warranting close monitoring of the patient’s cardiac function following discharge.

In conclusion, amphetamine use might induce life-threatening cardiovascular conditions that could have important diagnostic, prognostic, and therapeutic implications.
